# Supplementary material for: Effects of Olea europaea L. Polyphenols on the Animal Welfare and Milk Quality in Dairy Cows
Source: Animals (Basel). 2023 Oct 15;13(20):3225. doi: 10.3390/ani13203225 (PMC10603655; doi:10.3390/ani13203225)
Supplement: Supplementary file 1 [file animals-13-03225-s001.zip › animals-2611711-supplementary.pdf]

Supplementary Materials

Table S1. Strategic Table showing the mean values of metabolic parameters reported in the figures 1-8.

Parameters	Parity Class	Experimental Time					
		T0	T1	T2	T3	T4	T5
Glucose (mg/dl)	P	64.12^{a}	56.04$^{a/b}$	42.89	49.38	37.37^{b}	36.03^{a}
	S	54.43^{b}	52.69^{a}	45.42	45.63	51.85^{a}	45.67^{b}
	PL	69.97^{a}	57.93^{b}	41.80	47.64	51.75^{a}	33.60^{a}
Triglycerides (mg/dl)	P	9.24^{b}	7.37	3.60	3.15	3.64	10.49
	S	6.89^{a}	5.71	4.99	3.52	5.29	11.27
	PL	6.55^{a}	6.08	2.96	3.09	3.38	9.96
fT3 (pg/ml)	P	3.75^{b}	4.36	3.47	4.09	3.42	3.77^{b}
	S	3.96^{a}	3.90	3.87	3.70	3.26	3.20^{a}
	PL	3.71^{a}	3.88	3.71	3.90	2.97	2.86^{a}
fT4 (pg/ml)	P	0.92	0.88	0.72	0.68^{b}	0.67^{b}	0.82^{a}
	S	0.87	0.80	0.77	0.82^{a}	0.98^{a}	0.69^{b}
	PL	0.90	0.93	0.79	0.79^{a}	1.18^{c}	0.86^{a}
17β-Estradiol (pg/ml)	P	64.78^{a}	50.45	68.16	77.81^{a}	60.29	148.54^{a}
	S	37.88^{b}	52.20	63.68	54.53^{b}	60.16	85.39^{b}
	PL	29.36^{c}	44.71	61.83	40.66^{c}	60.78	125.27^{a}
ACTH (pg/ml)	P	19.37^{a}	19.96^{a}	18.24^{a}	18.72^{a}	22.92^{a}	25.45^{a}
	S	24.03^{a}	26.07^{b}	26.55^{b}	29.24^{b}	29.93^{a}	31.09^{a}
	PL	35.54^{b}	37.49^{c}	38.82^{c}	36.09^{c}	37.94^{b}	43.70^{b}
Cortisol (ng/ml)	P	6.33^{a}	4.61^{a}	4.94^{a}	4.74	5.07^{a}	10.29^{b}
	S	13.23^{b}	13.68^{b}	11.13^{b}	10.03^{b}	8.19^{a}	17.31^{a}
	PL	20.03^{c}	19.03^{c}	16.73^{c}	16.64^{c}	18.66^{b}	23.30^{a}
Prolactin (ng/ml)	P	1.55	1.43	1.63	1.72	2.03	2.46
	S	2.19	2.44	2.37	2.80	2.89	2.50
	PL	1.99	2.90	2.33	2.39	2.70	2.84
Oxytocin (pg/ml)	P	40.92^{a}	32.04^{b}	25.28^{a}	19.78	19.54	23.82$^{a/b}$
	S	33.47^{a}	22.80^{a}	25.64^{a}	20.85	20.75	27.00^{a}
	PL	46.76^{a}	20.08^{a}	17.73^{b}	22.78	21.35	19.37^{b}

Table S2. Summary results Two-way Anova (Tukey test) of milk fatty acids

Summary results Two-way ANOVA

Tukey's multiple comparisons test

C 14:0

P	Statistical Significance	S	Statistical Significance	PL	Statistical Significance
T0 vs. T1	ns	T0 vs. T1	***	T0 vs. T1	***
T0 vs. T2	ns	T0 vs. T2	***	T0 vs. T2	***
T0 vs. T3	ns	T0 vs. T3	***	T0 vs. T3	***
T0 vs. T4	*	T0 vs. T4	***	T0 vs. T4	***
T0 vs. T5	**	T0 vs. T5	***	T0 vs. T5	***
T1 vs. T2	ns	T1 vs. T2	***	T1 vs. T2	ns
T1 vs. T3	ns	T1 vs. T3	***	T1 vs. T3	***
T1 vs. T4	ns	T1 vs. T4	***	T1 vs. T4	***
T1 vs. T5	*	T1 vs. T5	***	T1 vs. T5	***
T2 vs. T3	ns	T2 vs. T3	***	T2 vs. T3	***
T2 vs. T4	ns	T2 vs. T4	***	T2 vs. T4	***
T2 vs. T5	ns	T2 vs. T5	***	T2 vs. T5	***
T3 vs. T4	ns	T3 vs. T4	***	T3 vs. T4	***
T3 vs. T5	ns	T3 vs. T5	***	T3 vs. T5	***
T4 vs. T5	ns	T4 vs. T5	**	T4 vs. T5	***

C 16:0

P	Statistical Significance	S	Statistical Significance	PL	Statistical Significance
T0 vs. T1	ns	T0 vs. T1	***	T0 vs. T1	***
T0 vs. T2	ns	T0 vs. T2	***	T0 vs. T2	***
T0 vs. T3	***	T0 vs. T3	***	T0 vs. T3	***
T0 vs. T4	***	T0 vs. T4	***	T0 vs. T4	***
T0 vs. T5	***	T0 vs. T5	***	T0 vs. T5	***
T1 vs. T2	ns	T1 vs. T2	***	T1 vs. T2	ns

T1 vs. T3	***	T1 vs. T3	***	T1 vs. T3	***
T1 vs. T4	***	T1 vs. T4	***	T1 vs. T4	***
T1 vs. T5	***	T1 vs. T5	***	T1 vs. T5	***
T2 vs. T3	***	T2 vs. T3	***	T2 vs. T3	***
T2 vs. T4	***	T2 vs. T4	***	T2 vs. T4	***
T2 vs. T5	***	T2 vs. T5	***	T2 vs. T5	***
T3 vs. T4	***	T3 vs. T4	ns	T3 vs. T4	***
T3 vs. T5	***	T3 vs. T5	*	T3 vs. T5	***
T4 vs. T5	*	T4 vs. T5	ns	T4 vs. T5	ns

C 16:1 n7t

P	Statistical Significance	S	Statistical Significance	PL	Statistical Significance
T0 vs. T1	***	T0 vs. T1	ns	T0 vs. T1	ns
T0 vs. T2	***	T0 vs. T2	**	T0 vs. T2	ns
T0 vs. T3	***	T0 vs. T3	***	T0 vs. T3	***
T0 vs. T4	***	T0 vs. T4	***	T0 vs. T4	***
T0 vs. T5	***	T0 vs. T5	***	T0 vs. T5	***
T1 vs. T2	***	T1 vs. T2	ns	T1 vs. T2	ns
T1 vs. T3	***	T1 vs. T3	***	T1 vs. T3	***
T1 vs. T4	***	T1 vs. T4	***	T1 vs. T4	***
T1 vs. T5	***	T1 vs. T5	***	T1 vs. T5	***
T2 vs. T3	***	T2 vs. T3	***	T2 vs. T3	**
T2 vs. T4	***	T2 vs. T4	***	T2 vs. T4	***
T2 vs. T5	***	T2 vs. T5	***	T2 vs. T5	***
T3 vs. T4	**	T3 vs. T4	***	T3 vs. T4	ns
T3 vs. T5	***	T3 vs. T5	***	T3 vs. T5	***
T4 vs. T5	***	T4 vs. T5	ns	T4 vs. T5	ns

C 16:1 n7

P	Statistical Significance	S	Statistical Significance	PL	Statistical Significance
T0 vs. T1	ns	T0 vs. T1	*	T0 vs. T1	ns
T0 vs. T2	*	T0 vs. T2	***	T0 vs. T2	ns
T0 vs. T3	**	T0 vs. T3	***	T0 vs. T3	***

T0 vs. T4	***	T0 vs. T4	***	T0 vs. T4	***
T0 vs. T5	***	T0 vs. T5	***	T0 vs. T5	***
T1 vs. T2	ns	T1 vs. T2	***	T1 vs. T2	ns
T1 vs. T3	ns	T1 vs. T3	***	T1 vs. T3	***
T1 vs. T4	ns	T1 vs. T4	***	T1 vs. T4	***
T1 vs. T5	*	T1 vs. T5	***	T1 vs. T5	***
T2 vs. T3	ns	T2 vs. T3	***	T2 vs. T3	*
T2 vs. T4	ns	T2 vs. T4	***	T2 vs. T4	***
T2 vs. T5	ns	T2 vs. T5	***	T2 vs. T5	***
T3 vs. T4	ns	T3 vs. T4	***	T3 vs. T4	**
T3 vs. T5	ns	T3 vs. T5	***	T3 vs. T5	***
T4 vs. T5	ns	T4 vs. T5	***	T4 vs. T5	**

C 18:0

P	Statistical Significance	S	Statistical Significance	PL	Statistical Significance
T0 vs. T1	***	T0 vs. T1	***	T0 vs. T1	***
T0 vs. T2	***	T0 vs. T2	***	T0 vs. T2	***
T0 vs. T3	***	T0 vs. T3	***	T0 vs. T3	***
T0 vs. T4	***	T0 vs. T4	***	T0 vs. T4	***
T0 vs. T5	***	T0 vs. T5	***	T0 vs. T5	***
T1 vs. T2	ns	T1 vs. T2	***	T1 vs. T2	**
T1 vs. T3	***	T1 vs. T3	***	T1 vs. T3	***
T1 vs. T4	***	T1 vs. T4	***	T1 vs. T4	***
T1 vs. T5	***	T1 vs. T5	***	T1 vs. T5	***
T2 vs. T3	***	T2 vs. T3	***	T2 vs. T3	***
T2 vs. T4	***	T2 vs. T4	***	T2 vs. T4	***
T2 vs. T5	***	T2 vs. T5	***	T2 vs. T5	***
T3 vs. T4	***	T3 vs. T4	***	T3 vs. T4	***
T3 vs. T5	***	T3 vs. T5	***	T3 vs. T5	***
T4 vs. T5	***	T4 vs. T5	***	T4 vs. T5	***

C 18:1 n9

P	Statistical Significance	S	Statistical Significance	PL	Statistical Significance

T0 vs. T1	ns	T0 vs. T1	***	T0 vs. T1	***
T0 vs. T2	ns	T0 vs. T2	***	T0 vs. T2	***
T0 vs. T3	***	T0 vs. T3	***	T0 vs. T3	***
T0 vs. T4	***	T0 vs. T4	***	T0 vs. T4	***
T0 vs. T5	***	T0 vs. T5	***	T0 vs. T5	***
T1 vs. T2	ns	T1 vs. T2	***	T1 vs. T2	**
T1 vs. T3	***	T1 vs. T3	***	T1 vs. T3	***
T1 vs. T4	***	T1 vs. T4	***	T1 vs. T4	***
T1 vs. T5	***	T1 vs. T5	***	T1 vs. T5	***
T2 vs. T3	***	T2 vs. T3	***	T2 vs. T3	***
T2 vs. T4	***	T2 vs. T4	***	T2 vs. T4	***
T2 vs. T5	***	T2 vs. T5	***	T2 vs. T5	***
T3 vs. T4	**	T3 vs. T4	***	T3 vs. T4	***
T3 vs. T5	***	T3 vs. T5	***	T3 vs. T5	***
T4 vs. T5	ns	T4 vs. T5	ns	T4 vs. T5	***

C 18:2 n6

P	Statistical Significance	S	Statistical Significance	PL	Statistical Significance
T0 vs. T1	ns	T0 vs. T1	***	T0 vs. T1	***
T0 vs. T2	ns	T0 vs. T2	***	T0 vs. T2	***
T0 vs. T3	**	T0 vs. T3	***	T0 vs. T3	***
T0 vs. T4	***	T0 vs. T4	***	T0 vs. T4	***
T0 vs. T5	***	T0 vs. T5	***	T0 vs. T5	***
T1 vs. T2	ns	T1 vs. T2	ns	T1 vs. T2	ns
T1 vs. T3	ns	T1 vs. T3	***	T1 vs. T3	***
T1 vs. T4	***	T1 vs. T4	***	T1 vs. T4	***
T1 vs. T5	***	T1 vs. T5	***	T1 vs. T5	***
T2 vs. T3	ns	T2 vs. T3	***	T2 vs. T3	***
T2 vs. T4	***	T2 vs. T4	***	T2 vs. T4	***
T2 vs. T5	***	T2 vs. T5	***	T2 vs. T5	***
T3 vs. T4	***	T3 vs. T4	***	T3 vs. T4	**
T3 vs. T5	***	T3 vs. T5	***	T3 vs. T5	***
T4 vs. T5	ns	T4 vs. T5	***	T4 vs. T5	***

C 18:3 n6

P	Statistical Significance	S	Statistical Significance	PL	Statistical Significance
T0 vs. T1	ns	T0 vs. T1	***	T0 vs. T1	***
T0 vs. T2	ns	T0 vs. T2	***	T0 vs. T2	***
T0 vs. T3	ns	T0 vs. T3	***	T0 vs. T3	***
T0 vs. T4	ns	T0 vs. T4	***	T0 vs. T4	***
T0 vs. T5	ns	T0 vs. T5	***	T0 vs. T5	***
T1 vs. T2	ns	T1 vs. T2	ns	T1 vs. T2	ns
T1 vs. T3	ns	T1 vs. T3	ns	T1 vs. T3	ns
T1 vs. T4	ns	T1 vs. T4	***	T1 vs. T4	***
T1 vs. T5	ns	T1 vs. T5	***	T1 vs. T5	***
T2 vs. T3	ns	T2 vs. T3	ns	T2 vs. T3	ns
T2 vs. T4	ns	T2 vs. T4	**	T2 vs. T4	***
T2 vs. T5	ns	T2 vs. T5	***	T2 vs. T5	***
T3 vs. T4	ns	T3 vs. T4	*	T3 vs. T4	***
T3 vs. T5	ns	T3 vs. T5	**	T3 vs. T5	***
T4 vs. T5	ns	T4 vs. T5	ns	T4 vs. T5	ns

C 20:1 n9

P	Statistical Significance	S	Statistical Significance	PL	Statistical Significance
T0 vs. T1	ns	T0 vs. T1	ns	T0 vs. T1	ns
T0 vs. T2	ns	T0 vs. T2	ns	T0 vs. T2	ns
T0 vs. T3	***	T0 vs. T3	ns	T0 vs. T3	***
T0 vs. T4	***	T0 vs. T4	ns	T0 vs. T4	***
T0 vs. T5	***	T0 vs. T5	ns	T0 vs. T5	***
T1 vs. T2	ns	T1 vs. T2	ns	T1 vs. T2	ns
T1 vs. T3	***	T1 vs. T3	ns	T1 vs. T3	***
T1 vs. T4	***	T1 vs. T4	ns	T1 vs. T4	***
T1 vs. T5	***	T1 vs. T5	ns	T1 vs. T5	***
T2 vs. T3	***	T2 vs. T3	ns	T2 vs. T3	***
T2 vs. T4	***	T2 vs. T4	ns	T2 vs. T4	***
T2 vs. T5	***	T2 vs. T5	ns	T2 vs. T5	***

T3 vs. T4	ns	T3 vs. T4	ns	T3 vs. T4	ns
T3 vs. T5	***	T3 vs. T5	ns	T3 vs. T5	**
T4 vs. T5	ns	T4 vs. T5	ns	T4 vs. T5	ns

C 18:3 n3

P	Statistical Significance	S	Statistical Significance	PL	Statistical Significance
T0 vs. T1	ns	T0 vs. T1	***	T0 vs. T1	***
T0 vs. T2	ns	T0 vs. T2	***	T0 vs. T2	***
T0 vs. T3	ns	T0 vs. T3	***	T0 vs. T3	***
T0 vs. T4	ns	T0 vs. T4	***	T0 vs. T4	***
T0 vs. T5	ns	T0 vs. T5	***	T0 vs. T5	***
T1 vs. T2	ns	T1 vs. T2	ns	T1 vs. T2	ns
T1 vs. T3	ns	T1 vs. T3	ns	T1 vs. T3	ns
T1 vs. T4	ns	T1 vs. T4	ns	T1 vs. T4	ns
T1 vs. T5	ns	T1 vs. T5	***	T1 vs. T5	**
T2 vs. T3	ns	T2 vs. T3	ns	T2 vs. T3	ns
T2 vs. T4	ns	T2 vs. T4	ns	T2 vs. T4	ns
T2 vs. T5	ns	T2 vs. T5	***	T2 vs. T5	*
T3 vs. T4	ns	T3 vs. T4	ns	T3 vs. T4	ns
T3 vs. T5	ns	T3 vs. T5	***	T3 vs. T5	*
T4 vs. T5	ns	T4 vs. T5	*	T4 vs. T5	ns

C 20:2 n6

P	Statistical Significance	S	Statistical Significance	PL	Statistical Significance
T0 vs. T1	**	T0 vs. T1	***	T0 vs. T1	***
T0 vs. T2	**	T0 vs. T2	***	T0 vs. T2	***
T0 vs. T3	***	T0 vs. T3	***	T0 vs. T3	***
T0 vs. T4	***	T0 vs. T4	***	T0 vs. T4	***
T0 vs. T5	***	T0 vs. T5	***	T0 vs. T5	***
T1 vs. T2	ns	T1 vs. T2	ns	T1 vs. T2	ns
T1 vs. T3	ns	T1 vs. T3	ns	T1 vs. T3	ns
T1 vs. T4	ns	T1 vs. T4	ns	T1 vs. T4	***
T1 vs. T5	ns	T1 vs. T5	ns	T1 vs. T5	***

T2 vs. T3	ns	T2 vs. T3	ns	T2 vs. T3	ns
T2 vs. T4	ns	T2 vs. T4	ns	T2 vs. T4	***
T2 vs. T5	ns	T2 vs. T5	ns	T2 vs. T5	***
T3 vs. T4	ns	T3 vs. T4	ns	T3 vs. T4	**
T3 vs. T5	ns	T3 vs. T5	ns	T3 vs. T5	***
T4 vs. T5	ns	T4 vs. T5	ns	T4 vs. T5	ns

C 20:3 n6

P	Statistical Significance	S	Statistical Significance	PL	Statistical Significance
T0 vs. T1	ns	T0 vs. T1	***	T0 vs. T1	***
T0 vs. T2	*	T0 vs. T2	***	T0 vs. T2	***
T0 vs. T3	**	T0 vs. T3	***	T0 vs. T3	***
T0 vs. T4	***	T0 vs. T4	***	T0 vs. T4	***
T0 vs. T5	***	T0 vs. T5	***	T0 vs. T5	***
T1 vs. T2	ns	T1 vs. T2	ns	T1 vs. T2	ns
T1 vs. T3	ns	T1 vs. T3	ns	T1 vs. T3	ns
T1 vs. T4	*	T1 vs. T4	ns	T1 vs. T4	*
T1 vs. T5	**	T1 vs. T5	ns	T1 vs. T5	**
T2 vs. T3	ns	T2 vs. T3	ns	T2 vs. T3	ns
T2 vs. T4	*	T2 vs. T4	ns	T2 vs. T4	*
T2 vs. T5	*	T2 vs. T5	ns	T2 vs. T5	*
T3 vs. T4	ns	T3 vs. T4	ns	T3 vs. T4	ns
T3 vs. T5	ns	T3 vs. T5	ns	T3 vs. T5	*
T4 vs. T5	ns	T4 vs. T5	ns	T4 vs. T5	ns

C 20:4 n6

P	Statistical Significance	S	Statistical Significance	PL	Statistical Significance
T0 vs. T1	*	T0 vs. T1	***	T0 vs. T1	***
T0 vs. T2	**	T0 vs. T2	***	T0 vs. T2	***
T0 vs. T3	**	T0 vs. T3	***	T0 vs. T3	***
T0 vs. T4	***	T0 vs. T4	***	T0 vs. T4	***
T0 vs. T5	***	T0 vs. T5	***	T0 vs. T5	***
T1 vs. T2	ns	T1 vs. T2	ns	T1 vs. T2	ns

T1 vs. T3	ns	T1 vs. T3	ns	T1 vs. T3	ns
T1 vs. T4	ns	T1 vs. T4	ns	T1 vs. T4	ns
T1 vs. T5	ns	T1 vs. T5	ns	T1 vs. T5	ns
T2 vs. T3	ns	T2 vs. T3	ns	T2 vs. T3	ns
T2 vs. T4	ns	T2 vs. T4	ns	T2 vs. T4	ns
T2 vs. T5	ns	T2 vs. T5	ns	T2 vs. T5	ns
T3 vs. T4	ns	T3 vs. T4	ns	T3 vs. T4	ns
T3 vs. T5	ns	T3 vs. T5	ns	T3 vs. T5	ns
T4 vs. T5	ns	T4 vs. T5	ns	T4 vs. T5	ns

C 24:0

P	Statistical Significance	S	Statistical Significance	PL	Statistical Significance
T0 vs. T1	***	T0 vs. T1	ns	T0 vs. T1	ns
T0 vs. T2	***	T0 vs. T2	ns	T0 vs. T2	ns
T0 vs. T3	***	T0 vs. T3	ns	T0 vs. T3	ns
T0 vs. T4	***	T0 vs. T4	ns	T0 vs. T4	ns
T0 vs. T5	***	T0 vs. T5	ns	T0 vs. T5	ns
T1 vs. T2	**	T1 vs. T2	ns	T1 vs. T2	ns
T1 vs. T3	***	T1 vs. T3	ns	T1 vs. T3	ns
T1 vs. T4	***	T1 vs. T4	ns	T1 vs. T4	ns
T1 vs. T5	***	T1 vs. T5	ns	T1 vs. T5	ns
T2 vs. T3	***	T2 vs. T3	ns	T2 vs. T3	ns
T2 vs. T4	***	T2 vs. T4	ns	T2 vs. T4	ns
T2 vs. T5	***	T2 vs. T5	ns	T2 vs. T5	ns
T3 vs. T4	***	T3 vs. T4	ns	T3 vs. T4	ns
T3 vs. T5	***	T3 vs. T5	ns	T3 vs. T5	ns
T4 vs. T5	**	T4 vs. T5	ns	T4 vs. T5	ns

C 20:5 n3

P	Statistical Significance	S	Statistical Significance	PL	Statistical Significance
T0 vs. T1	ns	T0 vs. T1	ns	T0 vs. T1	***
T0 vs. T2	ns	T0 vs. T2	ns	T0 vs. T2	***
T0 vs. T3	ns	T0 vs. T3	ns	T0 vs. T3	***

T0 vs. T4	ns	T0 vs. T4	ns	T0 vs. T4	***
T0 vs. T5	ns	T0 vs. T5	*	T0 vs. T5	***
T1 vs. T2	ns	T1 vs. T2	ns	T1 vs. T2	ns
T1 vs. T3	ns	T1 vs. T3	ns	T1 vs. T3	ns
T1 vs. T4	ns	T1 vs. T4	ns	T1 vs. T4	ns
T1 vs. T5	ns	T1 vs. T5	ns	T1 vs. T5	*
T2 vs. T3	ns	T2 vs. T3	ns	T2 vs. T3	ns
T2 vs. T4	ns	T2 vs. T4	ns	T2 vs. T4	ns
T2 vs. T5	ns	T2 vs. T5	ns	T2 vs. T5	ns
T3 vs. T4	ns	T3 vs. T4	ns	T3 vs. T4	ns
T3 vs. T5	ns	T3 vs. T5	ns	T3 vs. T5	ns
T4 vs. T5	ns	T4 vs. T5	ns	T4 vs. T5	ns

C 24:1 n9

P	Statistical Significance	S	Statistical Significance	PL	Statistical Significance
T0 vs. T1	ns	T0 vs. T1	ns	T0 vs. T1	ns
T0 vs. T2	ns	T0 vs. T2	**	T0 vs. T2	ns
T0 vs. T3	ns	T0 vs. T3	***	T0 vs. T3	ns
T0 vs. T4	***	T0 vs. T4	***	T0 vs. T4	***
T0 vs. T5	***	T0 vs. T5	***	T0 vs. T5	***
T1 vs. T2	ns	T1 vs. T2	ns	T1 vs. T2	ns
T1 vs. T3	ns	T1 vs. T3	*	T1 vs. T3	ns
T1 vs. T4	***	T1 vs. T4	***	T1 vs. T4	*
T1 vs. T5	***	T1 vs. T5	***	T1 vs. T5	***
T2 vs. T3	ns	T2 vs. T3	ns	T2 vs. T3	ns
T2 vs. T4	*	T2 vs. T4	*	T2 vs. T4	*
T2 vs. T5	**	T2 vs. T5	**	T2 vs. T5	***
T3 vs. T4	ns	T3 vs. T4	ns	T3 vs. T4	ns
T3 vs. T5	ns	T3 vs. T5	ns	T3 vs. T5	***
T4 vs. T5	ns	T4 vs. T5	ns	T4 vs. T5	ns

C 22:6 n3

P	Statistical Significance	S	Statistical Significance	PL	Statistical Significance

T0 vs. T1	ns	T0 vs. T1	***	T0 vs. T1	ns
T0 vs. T2	ns	T0 vs. T2	***	T0 vs. T2	ns
T0 vs. T3	ns	T0 vs. T3	***	T0 vs. T3	ns
T0 vs. T4	*	T0 vs. T4	***	T0 vs. T4	ns
T0 vs. T5	***	T0 vs. T5	***	T0 vs. T5	*
T1 vs. T2	ns	T1 vs. T2	ns	T1 vs. T2	ns
T1 vs. T3	ns	T1 vs. T3	ns	T1 vs. T3	ns
T1 vs. T4	*	T1 vs. T4	ns	T1 vs. T4	ns
T1 vs. T5	**	T1 vs. T5	ns	T1 vs. T5	ns
T2 vs. T3	ns	T2 vs. T3	ns	T2 vs. T3	ns
T2 vs. T4	*	T2 vs. T4	ns	T2 vs. T4	ns
T2 vs. T5	**	T2 vs. T5	ns	T2 vs. T5	ns
T3 vs. T4	ns	T3 vs. T4	ns	T3 vs. T4	ns
T3 vs. T5	ns	T3 vs. T5	ns	T3 vs. T5	ns
T4 vs. T5	ns	T4 vs. T5	ns	T4 vs. T5	ns

SFA

P	Statistical Significance	S	Statistical Significance	PL	Statistical Significance
T0 vs. T1	*	T0 vs. T1	***	T0 vs. T1	***
T0 vs. T2	***	T0 vs. T2	***	T0 vs. T2	***
T0 vs. T3	***	T0 vs. T3	***	T0 vs. T3	***
T0 vs. T4	***	T0 vs. T4	***	T0 vs. T4	***
T0 vs. T5	***	T0 vs. T5	***	T0 vs. T5	***
T1 vs. T2	ns	T1 vs. T2	***	T1 vs. T2	ns
T1 vs. T3	***	T1 vs. T3	***	T1 vs. T3	***
T1 vs. T4	***	T1 vs. T4	***	T1 vs. T4	***
T1 vs. T5	***	T1 vs. T5	***	T1 vs. T5	***
T2 vs. T3	***	T2 vs. T3	***	T2 vs. T3	***
T2 vs. T4	***	T2 vs. T4	***	T2 vs. T4	***
T2 vs. T5	***	T2 vs. T5	***	T2 vs. T5	***
T3 vs. T4	**	T3 vs. T4	***	T3 vs. T4	***
T3 vs. T5	***	T3 vs. T5	***	T3 vs. T5	***
T4 vs. T5	ns	T4 vs. T5	ns	T4 vs. T5	***

MUFA

P	Statistical Significance	S	Statistical Significance	PL	Statistical Significance
T0 vs. T1	***	T0 vs. T1	***	T0 vs. T1	***
T0 vs. T2	***	T0 vs. T2	***	T0 vs. T2	***
T0 vs. T3	***	T0 vs. T3	***	T0 vs. T3	***
T0 vs. T4	***	T0 vs. T4	***	T0 vs. T4	***
T0 vs. T5	***	T0 vs. T5	***	T0 vs. T5	***
T1 vs. T2	***	T1 vs. T2	***	T1 vs. T2	***
T1 vs. T3	***	T1 vs. T3	***	T1 vs. T3	***
T1 vs. T4	***	T1 vs. T4	***	T1 vs. T4	***
T1 vs. T5	***	T1 vs. T5	***	T1 vs. T5	***
T2 vs. T3	***	T2 vs. T3	***	T2 vs. T3	***
T2 vs. T4	***	T2 vs. T4	***	T2 vs. T4	***
T2 vs. T5	***	T2 vs. T5	***	T2 vs. T5	***
T3 vs. T4	***	T3 vs. T4	***	T3 vs. T4	***
T3 vs. T5	***	T3 vs. T5	***	T3 vs. T5	***
T4 vs. T5	***	T4 vs. T5	***	T4 vs. T5	***

PUFA

P	Statistical Significance	S	Statistical Significance	PL	Statistical Significance
T0 vs. T1	***	T0 vs. T1	***	T0 vs. T1	***
T0 vs. T2	***	T0 vs. T2	***	T0 vs. T2	***
T0 vs. T3	***	T0 vs. T3	***	T0 vs. T3	***
T0 vs. T4	***	T0 vs. T4	***	T0 vs. T4	***
T0 vs. T5	***	T0 vs. T5	***	T0 vs. T5	***
T1 vs. T2	ns	T1 vs. T2	ns	T1 vs. T2	***
T1 vs. T3	***	T1 vs. T3	***	T1 vs. T3	***
T1 vs. T4	***	T1 vs. T4	***	T1 vs. T4	***
T1 vs. T5	***	T1 vs. T5	***	T1 vs. T5	***
T2 vs. T3	***	T2 vs. T3	***	T2 vs. T3	***
T2 vs. T4	***	T2 vs. T4	***	T2 vs. T4	***
T2 vs. T5	***	T2 vs. T5	***	T2 vs. T5	***

T3 vs. T4	***	T3 vs. T4	***	T3 vs. T4	***
T3 vs. T5	***	T3 vs. T5	***	T3 vs. T5	***
T4 vs. T5	*	T4 vs. T5	***	T4 vs. T5	***

Abbreviations: P, Primiparous; S, Secondiparous; PL, Pluriparous.
Statisical significance ***p<0.001; **p<0.01; *p<0.05
